# Expression Analysis of *MYC* Genes from *Tamarix hispida* in Response to Different Abiotic Stresses

**DOI:** 10.3390/ijms13021300

**Published:** 2012-01-25

**Authors:** Xiaoyu Ji, Yucheng Wang, Guifeng Liu

**Affiliations:** State Key Laboratory of Tree Genetics and Breeding, Northeast Forestry University, 26 Hexing Road, 150040 Harbin, China; E-Mails: jixy0219@yahoo.com.cn (X.J.); wangyucheng1029@yahoo.com.cn (Y.W.)

**Keywords:** *MYC* gene, gene expression, *Tamarix hispida*, abiotic stresses

## Abstract

The *MYC* genes are a group of transcription factors containing both bHLH and ZIP motifs that play important roles in the regulation of abscisic acid (ABA)-responsive genes. In the present study, to investigate the roles of *MYC* genes under NaCl, osmotic and ABA stress conditions, nine *MYC* genes were cloned from *Tamarix hispida*. Real-time reverse-transcriptase (RT)-PCR showed that all nine *MYC* genes were expressed in root, stem and leaf tissues, but that the levels of the transcripts of these genes in the various tissues differed notably. The *MYC* genes were highly induced in the roots in response to ABA, NaCl and osmotic stresses after 3 h; however, in the stem and leaf tissues, *MYC* genes were highly induced only when exposed to these stresses for 6 h. In addition, most of these *MYC* genes were highly expressed in roots in comparison with stems and leaves. Furthermore, the *MYC* genes were more highly induced in roots than in stem and leaf tissues, indicating that these genes may play roles in stress responses mainly in the roots rather than the stems and leaves. The results of this present study suggest that *MYC*s are involved in salt and osmotic stress tolerances and are controlled by the ABA signal transduction pathway.

## 1. Introduction

MYCs (myelocytomatosis proteins) are a group of transcription factors found in plants and animals [1]. MYC2, which is allelic to Jasmonate insensitive 1 (JAI1/JIN1), was firstly identified from a mutant screen for reduced sensitivity to jasmonic acid (JA) [2]. Previous studies have shown that MYC2 plays an important role in the regulation of JA- and abscisic acid (ABA)-responsive genes [3–6]. MYC family members belong to a group of bHLH proteins that contain both bHLH and ZIP motifs [7]. The presence of both ZIP and bHLH motifs in a protein determine its specificity and affinity for sequence-specific DNA binding and can facilitate the formation of various hetero- and homodimers [8]. Through the interaction of their two amphipathic helices, MYCs can form homodimers or heterodimers [1,9]. Many MYC transcription factors bind to a consensus hexanucleotide sequence called the E-box (CANNTG) [10]. E-boxes can be divided into several types based on the identity of the two central bases in the sequence, and these include the G-box sequence “CACGTG” and the G-box-related motif “CACATG” [11–13]. The MYC2 transcription factor can bind with both the G-box-related motif [14,15] and the G-box sequence [16]. MYCs are involved in various processes, including the biosynthesis of anthocyanins, Tryptophan (Trp), proanthocyanidins and flavonoids; controlling the development of the trichome, carpel margin tissues, embryonic epidermis and root hairs; regulating floral initiation, the formation of the ER body, seed germination, stomatal differentiation, and endosperm breakdown; certain roles in the signaling pathways of jasmonate, ABA, phytochrome-mediated light, and gibberellin; and finally, mediating tolerances to biotic and abiotic stresses [17–21]. Gene expression analysis is an important way to elucidate the biological functions of certain genes. The genes responding to abiotic stresses are most likely to be involved in stress responses and tolerance. MYC transcription factors are involved in many biological processes, including important roles in the regulation of abiotic stress tolerance. However, there are few reports concerning the expression patterns of MYCs in response to different abiotic stresses.

Salt and drought are common adverse environmental factors and these have greatest impacts on plant productivity. Therefore, there is interest in selectively breeding plants that are tolerant to high salt and drought stresses. Studying plant tolerance to salty and arid soil at the molecular level may provide valuable information for improving salt and drought tolerances of plants by introducing targeted molecular breeding methods. *Tamarisk* (*Tamarix hispida*) is a shrub that is well adapted to arid, soda or saline soils. The ability of *T. hispida* to thrive in arid and saline soils indicates that this species has molecular and physiological systems that enable it to adapt and tolerate these stressful conditions, making it a desirable species for investigations into salt and drought tolerance in plants.

In this present study, nine unique *MYC* genes were cloned from *T. hispida*, and phylogenetic analysis was performed to uncover the genetic relationships between these genes. To elucidate the biological functions of the MYCs in responding to abiotic stresses, time-course expression of each gene was assessed in root, stem and leaf tissues during exposure to salt and drought stresses and the exogenous application of ABA. This study provides further insights into the roles of MYCs in abiotic stress tolerance in plants.

## 2. Results

### 2.1. Identification and Bioinformatics Analysis of *MYC* Genes from T. hispida

In total, nine unique *MYC* genes were identified from transcriptomes of *T. hispida*. These genes were designated as *ThMYC1* to *ThMYC9*, and these sequences have been deposited in the GenBank under accession numbers JN166785 to JN166790 and JN166792 to JN166794 (Table 1).

**Figure 1:**
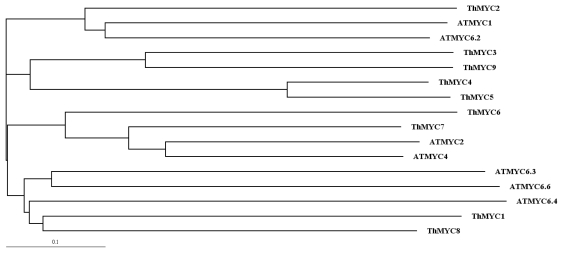
Phylogenetic analysis of the nine *ThMYC*s and seven *ATMYC*s : *ThMYC1* (JN166785), *ThMYC2* (JN166786), *ThMYC3* (JN166787), *ThMYC4* (JN166788), *ThMYC5* (JN166789), *ThMYC6* (JN166790), *ThMYC7* (JN166792), *ThMYC8* (JN166793) *ThMYC9* (JN166794), *ATMYC1* (AT4G00480), *ATMYC2*(AT1G32640), *ATMYC4* (AT4G17880), *ATMYC6.2* (AT5G41315), *ATMYC6.3* (AT5G41320), *ATMYC6.4* (AT5G41330) and *ATMYC6.6* (AT5G41350), The GenBank accession number of each *ThMYC* is shown in parentheses.

Among the nine unique *ThMYCs* identified, five had full open reading frames (ORFs) that encoded deduced polypeptides of 160 to 492 amino acids in length, with predicted MWs of 17.89 to 55.06 kDa and pI values of 4.77 to 7.67 (Table 2). The phylogenetic relationships between these *ThMYCs* were deduced from aligned sequences. The phylogenetic tree showed that these nine ThMYCs formed into three main subgroups: subgroup 1 contained *ThMYC1*, *ThMYC9*, *ThMYC2*, *ThMYC6* and *ThMYC5*; subgroup 2 contained *ThMYC3*, *ThMYC4* and *ThMYC8*; and subgroup 3 that contained *ThMYC7* only. *ThMYC7* showed similar genetic distances to subgroups 1 and 2 (Figure 1).

### 2.2. Relative Abundances of ThMYCs in Root, Stem and Leaf tissues

The relative abundances of the nine *ThMYCs* were determined by calculating CT values for each *ThMYC* in the leaves, stems and roots under normal growth conditions following real-time RT-PCR. The *ThMYC3* gene with the lowest expression level in roots (*i.e.*, highest delta-delta CT value) was used as a calibrator (designated as 1.0) to determine relative gene expression levels. Relative gene expression levels were log2 transformed and these data are shown in Table 3. There were notable differences in the abundances of the nine *ThMYCs* expressed in each tissue, particularly in the root tissues. The greatest differences in transcript abundances of the *ThMYCs* when cultivated under normal growth conditions were 511,604-fold in roots, 9.3-fold in stems and 12.7-fold in leaves in the *ThMYC1. ThMYC3* in root tissue was the transcript of lowest abundance, while *ThMYC4* was the gene of lowest abundance in stems and leaves.

### 2.3. Expression Patterns of ThMYCs in Response to Different Stresses

The molecular function of MYCs in leaves, stems and roots was determined by examining the expression profiles of the nine *ThMYCs* in response to different abiotic stresses using real-time RT-PCR. The genes were clustered according to similarities in their expression profiles for different treatments and after different treatment times. The clustering observed demonstrated a similar pattern as seen in the phylogenetic tree.

### 2.4. Expression Patterns of ThMYCs in Response to NaCl Stress

**Figure 2:**
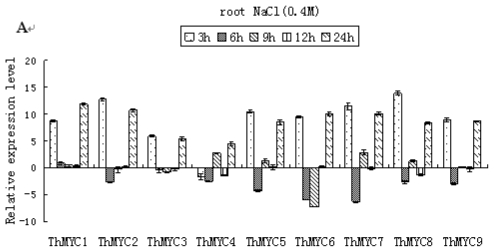

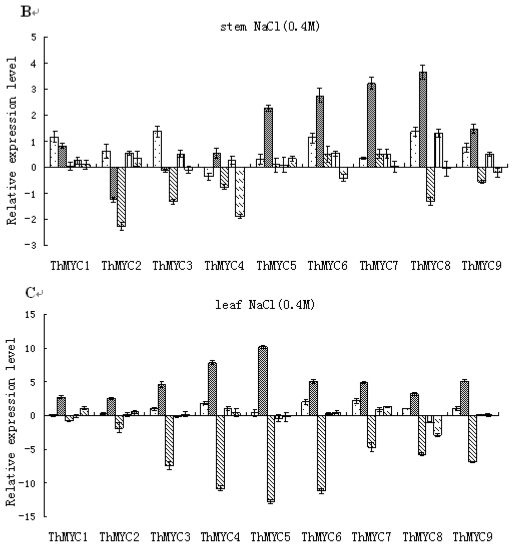
Time-course expression and hierarchical cluster analysis of *ThMYC*s in response to NaCl stress. Relative gene expression level was log_2_ transformed: >0, up-regulation; =0, no change in regulation; <0, down-regulation. (**A**–**C**): expression of *ThMYC*s in roots, stems and leaves, respectively.

The expression patterns of the *ThMYCs* in response to NaCl treatment were investigated. In the roots, all the *ThMYCs*, except *ThMYC4*, displayed similar expression patterns. They were highly induced by NaCl stress at 3 h, but then their transcription levels decreased at 6, 9 and 12 h. At 24 h, the *ThMYCs* were highly expressed (increases of up to 128-fold), suggesting that the *ThMYCs* (except *ThMYC4*) play roles in NaCl stress tolerance in roots (Figure 2A). In stem tissues, *ThMYC5*, *ThMYC6*, *ThMYC7*, *ThMYC8* and *ThMYC9* displayed similar expression patterns. They were highly induced by NaCl stress and reached peak expression levels at 6 h. *ThMYC1* was induced after 3 h of NaCl stress, but the expression of this gene was not significantly different from expression in the control stem tissues at the other time points. *ThMYC3* was induced after 3 h of NaCl treatment, but was highly down-regulated at 9 h. *ThMYC2* and *ThMYC4* were generally down-regulated after NaCl stress treatment (Figure 2B). In leaves, the *ThMYCs* displayed similar expression profiles. They were highly induced by NaCl stress at 6 h, before being down-regulated at 9 h. At subsequent time points, *ThMYC* expression levels were relatively similar to the controls, except *ThMYC8* that showed a down regulation at 24 h (Figure 2C).

### 2.5. Expression Patterns of ThMYCs in Response to PEG Stress

**Figure 3:**
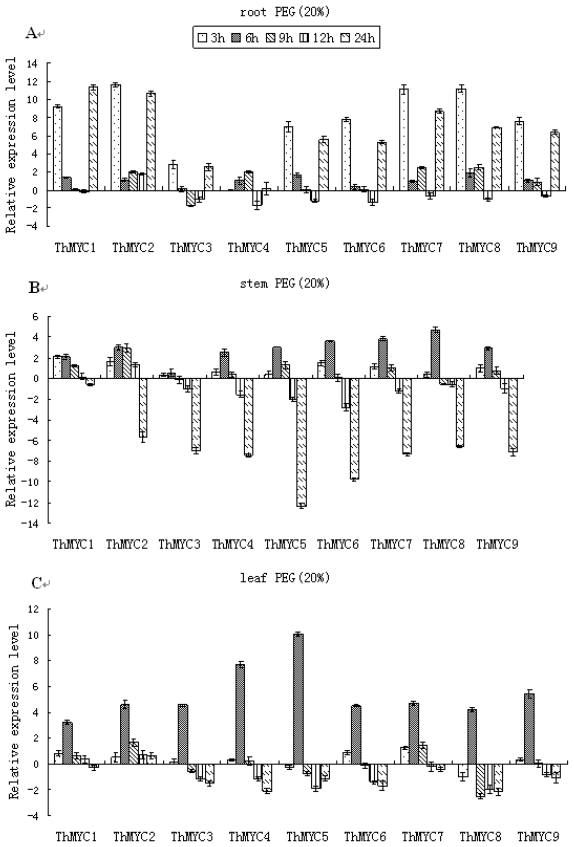
Time-course expression and hierarchical cluster analysis of *ThMYC*s in response to PEG stress. Relative gene expression level was log_2_ transformed: >0, up-regulation; =0, no change in regulation; <0, down-regulation. (**A**–**C**): expression of *ThMYC*s in roots, stems and leaves, respectively.

In roots, as with NaCl stress, all of the *ThMYC* genes (except for *ThMYC4*) were highly induced by PEG stress at 3 and 24 h, and generally transcript levels peaked at 3 h. However, at the other time points tested, the expression of these genes in the roots did not differ notably from the controls (Figure 3A). In stems, *ThMYC1* was significantly induced by PEG stress at 3, 6 and 9 h, but expression was not significantly different from the controls at later time points. The other *ThMYCs* generally showed similar expression patterns in which the expression peaked at 6 h of PEG stress before decreasing to their lowest levels at 24 h. In other words, the *ThMYCs* were down-regulated at 24 h. (Figure 3B). In leaves, *ThMYC1* was significantly up-regulated by PEG treatment at 3, 6, 9 and 12 h, but expression did not differ significantly from the control at 24 h. The other *ThMYCs* genes were highly up-regulated by PEG at 6 h (typically peak expression levels), but at the other time points their expression did not differ significantly from the control or were even down-regulated (Figure 3C).

### 2.6. Expression Patterns of ThMYCs in Response to ABA Treatment

**Figure 4:**
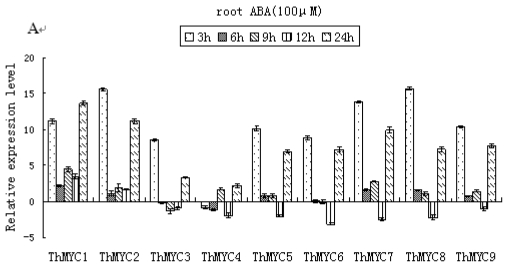

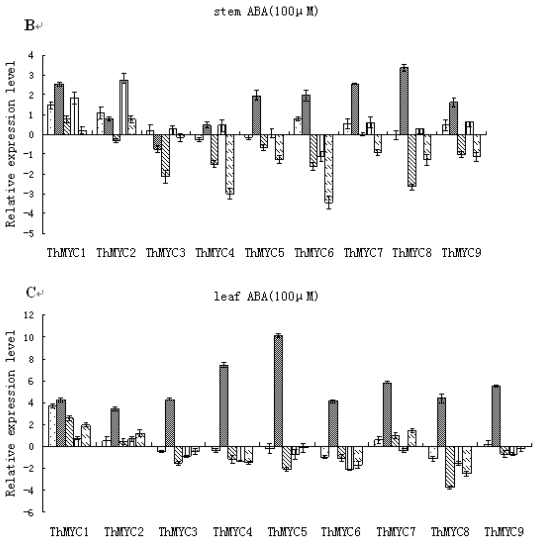
Time-course expression and hierarchical cluster analysis of *ThMYC*s in response to ABA treatment. Relative gene expression level was log_2_ transformed: >0, up-regulation; =0, no change in regulation; <0, down-regulation. (**A**–**C**): expression of *ThMYC*s in roots, stems and leaves, respectively.

Real-time RT-PCR results demonstrated that all the *ThMYCs* in the roots (except *ThMYC4*) were highly induced by ABA exposure at 3 and 24 h; indeed, peak expression levels generally occurred at 3 h (except *ThMYC1*). Nevertheless, the expression of these genes was much lower at the other time points examined compared with 3 and 24 h (Figure 4A). In stems, *ThMYC5*, *ThMYC6*, *ThMYC7*, *ThMYC8* and *ThMYC9* showed similar expression patterns, and these genes were significantly induced 6 h after ABA treatment, but were down-regulated or did not differ significantly from controls at the other time points investigated. While *ThMYC1* and *ThMYC2* were generally up-regulated by ABA treatment, the expression of *ThMYC3* and *ThMYC4* was significantly down-regulated or did not differ significantly from the controls (Figure 4B). In leaves, all of the *ThMYCs* were induced by ABA treatment and transcript levels peaked at 6 h. At the other time points, except for *ThMYC1*, the expression levels of all of the other genes were similar to the controls or were significantly down-regulated. Indeed, *ThMYC1* was significantly up-regulated by ABA treatment at 3, 6, 9 and 24 h (Figure 4C).

## 3. Discussion

MYCs are involved in stress responses and these transcription factors have been found to regulate the expression of ABA-responsive genes [22]. In the present study, we cloned nine *MYC* genes from *T. hispida*. To investigate the roles of these *ThMYCs* in stress responses, their expression patterns were analyzed in response to salt, osmotic and ABA stresses using real-time RT-PCR. Our data showed that all nine *ThMYCs* were expressed in leaves, stems and roots, but that they exhibited tissue-specific expression patterns in response to salt, osmotic and ABA stresses. These findings highlight the need to investigate the expression of *ThMYCs* in different plant organs in order to reveal their detailed roles in stress responses.

The *ThMYCs* showed notable differences in abundances between leaf and stem tissues under normal growth conditions (Table 3). Except for *ThMYC3*, all of the *ThMYCs* were highly expressed in roots compared with the stems and leaves, especially *ThMYC4* and *ThMYC8*, whose expression was mainly found in the roots (Table 3). In addition, *ThMYCs* were more highly induced in roots than in the stems and leaves in response to salt and osmotic stresses and ABA treatment. These results suggest that the *ThMYCs* play their roles in stress responses mainly in the roots rather than in stems and leaves. The abundance of *ThMYC3* was lower than the other *ThMYC* genes, but its transcript was most abundant in stem tissues than in the roots and leaves. Moreover, in response to the various stresses, *ThMYC3* transcripts were highly increased in the roots and leaves compared with the stems. These results indicate that *ThMYC3* mainly functions in the roots and leaves under conditions of stress.

In roots, all of the *ThMYCs* (except for *ThMYC4*) were highly induced by NaCl, PEG and ABA treatments. Interestingly, our results showed that in general the *ThMYCs* displayed similar expression patterns under salt, osmotic and ABA stress conditions, specifically that these genes were highly induced at 3 and 24 h compared with the other time points. Thus, the *ThMYCs* were highly activated at 3 and 24 h, and these could be the times at which the *ThMYCs* may regulate their target genes to mediate stress tolerance. Furthermore, all of the *ThMYCs* (except for *ThMYC4*) displayed similar expression patterns under salt and osmotic stresses, which suggests that these ThMYCs may play similar roles in salt and osmotic stress responses. The expression of *ThMYC4* differed notably from the other *ThMYCs* in the roots and this gene was not highly regulated by salt, osmotic and ABA stresses, which suggests that it may not play an important role in stress responses in root tissue.

In stems, most of the *ThMYCs* were up-regulated by salt and osmotic stresses and ABA treatment after 6 h, and their expression peaked at this time point. Similarly, In the leaves, all of the *ThMYCs* were highly up-regulated by ABA exposure and salt and osmotic stresses after 6 h. These results suggest that the *ThMYCs* may regulate their target genes in stems and leaves for adaptation to stressful conditions 6 h after stress treatment.

Interestingly, our results showed that all of the *ThMYCs* were highly induced by ABA exposure and salt and osmotic stresses in the roots at 3 h; however, in the stems and leaves, they were highly induced only after stress for 6 h. This is probably because the root is the first plant organ to perceive the stress signal, and it presumably takes time to transduce stress signals to the stems and leaves to trigger the expression of the *ThMYCs* in those tissues. Our results showed that the *ThMYCs* were highly induced at early stress period (stress for 3 or 6 h) and then were inhibited. Consistent with our results, some reports also showed that the transcriptional factors were highly induced at early stress period and then decreased [23–25]. This phenomenon may be due to the reason that plants perceive the stress environment and produced the stress signal to trig the expression of *MYCs*, and the induction of *MYCs* will regulate the expression of their targets genes related stress response to adapt stress environment; when the regulation of *MYC* target genes was completed, the expression of *MYCs* will be decreased.

MYC proteins are synthesized only after endogenous levels of ABA accumulate, suggesting that these proteins play roles in the latter stages of stress responses [26]. The ThMYCs function as transcriptional activators in the ABA signal transduction pathway under stress conditions in plants [27]. Our results showed that all of the *ThMYCs* were up-regulated by ABA in the roots and leaves. Moreover, *ThMYCs* were highly up-regulated by salt and osmotic stresses. These results confirm that *ThMYCs* are stress responsive genes and that they belong to an ABA-dependent signaling pathway. Our results also showed that the *ThMYCs* were more highly induced in roots and leaves than in the stems, suggesting that they may play roles in stress responses mainly in roots and leaves rather than in the stems. Further, these findings may also imply that stress responses occur mainly in roots and leaves. That *ThMYC3* and *ThMYC4* were not up-regulated in response to ABA in the stems but were up-regulated in leaves and roots indicates that there is tissue specificity with respect to ABA induction of *ThMYC* gene transcription.

In general, our results showed that *ThMYC5*, *ThMYC6*, *ThMYC7*, *ThMYC8* and *ThMYC9* displayed similar expression in response to certain stress treatments, suggesting that these genes may be involved in the same gene expression regulatory networks in response to stress. Conversely, *ThMYC1*, *ThMYC2*, *ThMYC3* and *ThMYC4* displayed different expression patterns in response to stress, suggesting that these genes may be involved in distinct gene regulation pathways.

## 4. Experimental Section

### 4.1. Plant Culture Conditions and Treatments

Seeds for propagation of the plant materials were harvested from *T. hispida* plants. The seeds were planted into pots containing a mixture of turf peat and sand (2:1, v/v) and these were well-watered and kept under controlled greenhouse conditions of 70–75% relative humidity, 14 h light/10 h dark, and an average temperature of 24 °C. Two-month-old seedlings were exposed to one of four different treatments for 3, 6, 9, 12 or 24 h: water (normal growth condition without stress; control), 0.4 M NaCl, 20% polyethylene glycol (PEG)-6000 or 100 μM ABA. After each treatment, root, stem, and leaf tissues from at least five seedlings were harvested. The harvested roots, stems or leaves from each seedling were pooled, frozen immediately in liquid nitrogen for RNA preparation, and then analyzed by real-time reverse-transcriptase (RT)-PCR.

### 4.2. Cloning and Analysis of *MYC* Family Genes from T. hispida

Four transcriptomes were created from root tissues of *T. hispida* treated with NaHCO_3_ for 0, 12, 24 and 48 h using Solexa technology. Totals of 66,300, 51,204, 51,634 and 56,355 tentative unigenes (TUGs) were generated from the 0, 12, 24 and 48 h libraries, respectively. These TUGs were assembled into 81344 non-redundant unigenes using TGI Clustering tools [28]. These non-redundant unigenes were subjected to BLASTX analysis against protein databases, NR and Swiss-Prot, to search for similarities. Unigenes with BLASTX E-values > 10^−5^ were discarded during functional annotation. MYC genes were identified during functional annotation of the non-redundant unigenes by BLASTX analysis.

### 4.3. Phylogenetic Analysis of MYC Sequences

The open reading frame (ORF) of each MYC was resolved using ORF Finder from NCBI [29]. MYCs with complete ORFs were subjected to further analysis. Phylogenetic reconstruction was carried out using ClustalX Version 1.81 and the neighbor-joining method [30]. Sequence identities between the MYCs were calculated using ClustalW2 [31]. Classification of the nine MYCs was performed according to the method of [32]. Molecular weight (MW) and isoelectric point (pI) predictions for each MYC was carried out using the Compute pI/Mw tool [33].

### 4.4. MYC Gene Expression Profiles in Response to Abiotic Stresses

Total RNA of each sample was extracted using the CTAB method [34], and then was treated with DNaseI (Promega) to remove any residual DNA. RNA concentration was measured using a BioPhotometer plus (Eppendorf, Germany). Approximately 1 μg of total RNA was reverse-transcribed to cDNA in a 10 μL volume using 1 μM of oligodeoxythymidine as primer, and the procedures of cDNA synthesis was following the PrimeScriptTM RT Reagent Kit protocol (TaKaRa Corp., Dalian, China). The synthesized cDNA was diluted to 100 μL with sterile water and used as template for real-time RT-PCR.

Real-time RT-PCR was performed in an MJ OpticonTM2 machine (Bio-Rad, Hercules, CA, USA). The β-actin, α-tubulin and β-tubulin genes were selected as internal controls to normalize the quantity of total RNA present in each reaction. The primers used for real-time RT-PCR are shown in Table 1. The reaction mixture (20 μL) contained 10 μL of SYBR Green Realtime PCR Master Mix (Toyobo), 0.5 μM each of forward and reverse primers, and 2 μL of cDNA template (equivalent to 100 ng of total RNA). For PCR amplification, all the primer pairs were used the same amplification procedure. The amplification was performed using the following cycling parameters: 94 °C for 30 s; 45 cycles of 94 °C for 12 s, 60 °C for 30 s and 72 °C for 40 s; and 1 s at 82 °C for plate reading. A melting curve was generated for each sample at the end of each run to assess the purity of the amplified products. Real-time RT-PCR was carried out in triplicate (technical repeats) to ensure the reproducibility of the results. Three biological repeats were performed on each treatment. Expression levels were calculated from the threshold cycle according to the delta-delta CT method [34]. Relative gene expression level was calculated as the transcription level under stress treatment divided by the transcription level of the control (*i.e.*, samples from plants grown under normal conditions and harvested at the same time). Relative gene expression levels were log_2_ transformed.

## 5. Conclusions

In conclusion, we have constructed expression profiles for nine *ThMYC* genes in different organs of *T. hispida* in response to salt and osmotic stresses and ABA treatment. It was shown that *ThMYCs* can be up-regulated by NaCl, PEG and ABA, indicating that *ThMYCs* are involved in salt and osmotic stress tolerance and are controlled by ABA. Also, these *ThMYCs* are more highly expressed and more highly induced by salt, PEG and ABA treatments in roots compared with stem and leaf tissues, suggesting that these genes may play roles in stress responses in the roots rather than in leaves and stems.

## Figures and Tables

**Table 1:** Primers used for real time RT-PCR.

Gene	GenBank Accession number	Forward Primers (5′-3′)	Reverse Primers (5′-3′)
*ThMYC1*	JN166785	AGGCTTAATGACAAGTTTGTGG	AGCGTATGCGGCTGGCATTGTT
*ThMYC2*	JN166786	GTATCCGGATATAGTTGAGCAG	GGATGCCATCAAGAGTTGATG
*ThMYC3*	JN166787	AATGGTAGTGGTAGAGTCGGTG	CTCATCATTAGGTCCTGACGAT
*ThMYC4*	JN166788	GAAGCGATTGAGGGAAGATGAT	CTTCACATACTCCACTGCTTCT
*ThMYC5*	JN166789	TTGAGTGGAAGCGTTGATGGGT	TATCACTAATTCTTGTCCTTCG
*ThMYC6*	JN166790	TGAGTACTTGGTAGCTAGCTCT	ATCATCATCATCAGAACCACTG
*ThMYC7*	JN166792	TAGGAACCGAAGTCTGGATCCT	GTATAGGTATACATACCAGAGT
*ThMYC8*	JN166793	ATGAAAACTCTTTACTCACAGC	TCCGACCCTACGCGTATGTGTC
*ThMYC9*	JN166794	TCAAGCTACTGATAGCCACAGT	TCGAATGTAGTTGGAGCAAGCT
Actin	FJ618517	AAACAATGGCTGATGCTG	ACAATACCGTGCTCAATAGG
α-tubulin	FJ618518	CACCCACCGTTGTTCCAG	ACCGTCGTCATCTTCACC
β-tubulin	FJ618519	GGAAGCCATAGAAAGACC	CAACAAATGTGGGATGCT

**Table 2:** Characteristics of the five *ThMYC*s with a full-length open reading frame (ORF).

Gene	cDNA Length (bp)	Mature Protein		

		Amino acid length	MW (kDa)	pI
*ThMYC1*	717	238	26.42	7.67
*ThMYC2*	1479	492	55.06	4.77
*ThMYC4*	483	160	17.89	5.96
*ThMYC5*	810	269	28.00	6.30
*ThMYC9*	906	301	31.69	6.19

**Table 3:** Relative abundances of *ThMYC*s in different tissues.

	Relative abundance		
	
Gene	Roots	Stem	Leaves
*ThMYC1*	511,603.5	9.257782	12.73741
*ThMYC2*	5248.417	2.206867	5.107675
*ThMYC3*	1	1	1
*ThMYC4*	1629.259	0.466624	0.877619
*ThMYC5*	321,620.5	2.7007	2.800466
*ThMYC6*	396,510.6	2.046748	3.219121
*ThMYC7*	85,284.74	1.304352	1.171752
*ThMYC8*	31,916.17	0.666803	1.085731
*ThMYC9*	14,050.09	1.613051	2.249142
